# Clinical and inflammatory factors associated with prevalent gout in a Brazilian hospital population

**DOI:** 10.1016/j.clinsp.2026.101083

**Published:** 2026-08-11

**Authors:** Jordana Dinorá de Lima, Leonel Witcoski Junior, André Guilherme Portela de Paula, Andressa Pacheco Czaikovski, Bruna Sadae Yuasa, Gabriela Yanaze Takamatsu, Caio Cesar Souza Smanioto, Maria Clara da Cruz Silva, Ederson Borges Francisco, Juliana Navarro Ueda Yaochite, Marco Antonio de Freitas Clementino, Felipe Fortino Verdan, Valéria Vidal, Cyntia Nancy Weçoski, Niels Olsen Saraiva Câmara, Fellype Barreto, Eduardo S. Paiva, Valderílio Feijó Azevedo, Bianca de Oliveira Cata-Preta, Tárcio Teodoro Braga

**Affiliations:** a Microbiology, Parasitology and Pathology Graduate Program, Universidade Federal do Parana (UFPR), Curitiba, PR, Brazil; b Medical Microbiology Graduate Program, Universidade Federal do Ceara (UFC), Fortaleza, CE, Brazil; c Complexo Hospital de Clínicas, Universidade Federal do Parana (UFPR/EBSERH), Curitiba, PR, Brazil; d Biomedical Sciences Institute IV, Universidade de São Paulo (USP), São Paulo, SP, Brazil; e Department of Internal Medicine, Rheumatology Unit, Universidade Federal do Parana (UFPR/EBSERH), Curitiba, PR, Brazil; f Department of Public Health, Universidade Federal do Parana (UFPR), Curitiba, PR, Brazil; g Biosciences and Biotechnology Graduate Program, Instituto Carlos Chagas (ICC/Fiocruz), Curitiba, PR, Brazil

**Keywords:** Sex factor, Alcohol, IL-6, Monocyte, Gout flare, Brazilian population

## Abstract

**Objective:**

Gout is a rheumatological disease characterized by the crystallization of uric acid (UA) in joints and tissues, which is believed to trigger inflammatory responses. This study aimed to characterize the inflammatory profile of gout patients in a Brazilian population and its potential influence on flare frequency.

**Methods:**

In this observational cross-sectional study, we analyzed 193 patients from the Complex of Clinics Hospital in Curitiba, Brazil, categorized into two groups: gout (*n* = 102) and non-gout (*n* = 91). Data were obtained through in-person surveys, consultation of medical records, and serum analyses. We evaluated anthropometric measurements, lifestyle habits, medication use, serum UA levels, and cytokine profiles.

**Results:**

Males were more prevalent in the gout group than in the non-gout group (*p* < 0.001). The gout group also showed higher consumption of uricogenic foods, including ethanol (*p* = 0.006), sugary drinks (*p* = 0.001), meat and seafood (*p* < 0.001), and processed meats (*p* = 0.034). UA levels were not correlated with cytokine presence and were not effective in distinguishing patients with low or high flare frequency (OR = 1.07; *p* = 0.6; AUC = 0.522). IL-6 levels alone modestly differentiated flare frequency (OR = 0.99; *p* = 0.8; AUC = 0.788), with improved discrimination when combined with monocyte counts (OR = 0.84; *p* = 0.029; AUC = 0.801).

**Conclusions:**

IL-6, particularly when combined with monocyte count, may be worth considering as a potential marker of gout flare frequency, enabling earlier identification of high-risk patients and supporting targeted interventions to improve disease management and outcomes.

## Introduction

Gout is an inflammatory disease caused by the crystallization of Uric Acid (UA) in joints and tissues, often leading to inflammatory responses.^1^^,^^2^ The incidence of gout varies across the globe, while Oceania reported a prevalence of 6.8% between 2008 and 2013, North America 3%‒4% between 2007 and 2008,^3^ and Europe 1% to 4% in data collected between 2003 and 2014.^4^ However, data on gout incidence in South America remain.^4^^,^^5^ Brazilian studies, in particular, highlight the need for further investigation in this area,^6^ especially given the projected 70% increase in gout cases over the next 30-years.^7^

In the context of gout pathogenesis, UA is reported as the most used biochemical analyte in clinical practice, particularly targeted at preventing gout-associated flares or tophi formation.^8^ However, the inflammatory response to UA varies widely: while some hyperuricemic patients show no signs of inflammation,^9^ others with normouricemia exhibit significant inflammation^10^ ‒ a phenomenon known as the hyperuricemia paradox. Similarly, although dietary modification, such as reduced intake of purine-rich foods, is clinically recommended to lower urate blood concentration, the diet alone is not directly correlated to uric acid presence,^11^ further highlighting the complexity of gout pathogenesis.

Additionally, cytokines have been described as important biomarkers for differentiating patients with gout from healthy ones.^12^ The immune response to UA may provide important insights into gout pathogenesis, particularly regarding the interaction between macrophages and neutrophils.^13^ In this process, macrophages are responsible for crystal phagocytosis and synthesis of cytokines and chemokines, while neutrophils amplify the inflammatory response and produce NETs, promoting crystal degradation.^14^ Moreover, impairment of these cells may influence the response to hyperuricemia.^15^ Therefore, the authors’ primary objective was to characterize the investigated Brazilian population, particularly regarding factors associated with gout, such as lifestyle habits. We also aimed to explore the potential of cytokines to identify patients more sensitive to the manifestation of gout, particularly to gout flares.

## Methods

### Selection of eligible patients

For this observational cross-sectional study, patients with scheduled appointments at Internal Medicine Department of the *Complexo do Hospital de Clinicas* (CHC-UFPR), especially from the Rheumatology and Nephrology units, with scheduled appointments, were invited to participate in an in-person survey (Supplementary Appendix 1) and blood collection. Exclusion criteria, obtained through medical chart review, included current infectious diseases, neoplasms, immunosuppressive therapy, recent organ transplant or renal replacement therapy, and dementia. We also collected retrospective data from all patients who had a scheduled appointment between August and December of 2021, met the inclusion criteria, and accepted the invitation. After that, a total of 139 patients were allocated to two groups: gout (*n* = 48), and non-gout (*n* = 91). Written free and informed Consent Forms were provided and signed by all participants. Additional laboratory and clinical data from 54 gout patients collected between 2017 and 2021 were included, resulting in a final sample of 193 patients. Importantly, the non-gout group was not comprised of healthy controls. Rather, these individuals were patients under care in the Rheumatology or Nephrology Units for various conditions, with the defining characteristic of lacking a gout diagnosis. For detailed information, please check Supplemental Table 1. The study was approved by the Research Ethics Committee (CEP CAAE ‒ 37,306,220.4.0000.0102 ‒ Resolutions n° 466/12 and 441/2011 ‒ National Health Council – Supplementary Appendix 2). All data is available upon request to the corresponding author.

### Gout and hyperuricemia classification

Individuals allocated to the G group were diagnosed according to the 2015 American College of Rheumatology and European League Against Rheumatism (ACR/EULAR) criteria.^15^ Hyperuricemia was defined in cases of serum Uric Acid (UA) above 7 mg/dL for men and above 6 mg/dL for women. Importantly, hyperuricemia was determined regardless of gout presence.

### Sample characteristics

The investigation of this population was structured around variables encompassing three main categories of variables: clinical and biochemical parameters, lifestyle habits, and serum inflammatory markers. For clinical and biochemical assessments, blood samples were collected from each participant immediately following recruitment. Serum uric acid was quantified from the serum fraction, while relative monocyte counts were determined from the cellular fraction, with laboratory analyses conducted by the Clinical Hospital Complex of the Federal University of Paraná (UFPR/EBSERH). Additional laboratory and clinical data were obtained through medical chart review and were restricted to measurements recorded within two years prior to blood collection. Lifestyle habits were self-reported during in-person interviews (Supplementary Appendix 1), along with the frequency of gout flares. Moreover, flares were categorized as high frequency (≥ 1 episode every 3-months) or low frequency (< 1 episode every 3-months).

### Estimation of inflammatory status

To assess inflammatory status in individuals from the study, we measured nine popular serum inflammatory markers ‒ including IFN-γ, IL-1β, IL-2, IL-6, IL-10, IL-12, IL-18, MCP-1, and TNF-α ‒ which were quantified using the Procartaplex Multiplex assay and Luminex xMAP technology, according to the manufacturer’s instructions. Briefly, magnetic beads pre-coated with capture antibodies were incubated overnight, followed by the addition of patient serum and standards. Detection involved biotinylated antibodies, Streptavidin-PE, and reading reagents, with washes between steps. Data were acquired on the MAGPIX® system (Merck, MA, USA).

### Statistical analysis

Data was analyzed using RStudio version 6.0.1.7628. Multivariate analysis was performed using binary logistic regression models with backward stepwise selection. Comparisons between the two groups (gout and non-gout) were performed using ANOVA, Fisher’s exact test, Pearson's Chi-squared test, Wilcoxon rank sum test, and Kruskal-Wallis rank sum test according to the nature of the variables analyzed, with Bonferroni correction applied where appropriate. Predictive models were further evaluated for accuracy using cross-validation, in which 80% of the sample was used for training and the remaining 20% for validation. We reported the prevalence of gout alongside a 95% confidence interval and considered a p-value < 0.05 statistically significant. Explanations about possible confounding are available in the Supplementary Material.

## Results

### Sample’s description

The sampled population was mainly composed of individuals younger than 65-years (*n* = 90, 53.4%), predominantly men (*n* = 149, 77.2%). Most of the sample did not engage in exercise (*n* = 146, 76.4%) or smoke (*n* = 167, 89.2%). Overall, the diet of the whole sampled population was perceived as healthy, with lower levels of ethanol, sausages, sugary drinks, and sugary food intake (Table 1).

**Table 1:** Demographic and clinical characteristics of the study population (*n* = 193). Measurement units ‒ Uric acid: mg/dL, IL-18: pg/mL, and MCP1: pg/mL.

	Level	Overall	Available data^a^
**Total sampling number**		193	
**Group (%)**	Non-Gout	91 (47.2)	193
	Gout	102 (52.8)	
**Age, mean (SD)**		63.49 (12.59)	193
**Sex (%)**	Female	44 (22.8)	193
	Male	149 (77.2)	
**Uric acid, mean (SD)**		7.07 (2.15)	191
**Color (%)**	White	93 (66.9)	139
	Brown	34 (24.5)	
	Black	10 (7.2)	
	Yellow	2 (1.4)	
**Body mass index, mean (SD)**		29.21 (5.19)	140
**Physical Activity frequency (%)**	Do not practice	146 (76.4)	191
	1‒2×/week	41 (21.5)	
	>2×/week	4 (2.1)	
**Alcoholic beverages consumption (%)**			191
	Do not consume	150 (78.5)	
	1‒2×/week	37 (19.4)	
	3‒4×/week	1 (0.5)	
	Everyday	3 (1.6)	
**Sugary Drinks consumption (%)**	Do not consume	114 (59.7)	191
	1‒2×/week	42 (22.0)	
	3‒4×/week	12 (6.3)	
	Everyday	23 (12.0)	
**Red meat consumption (%)**	Do not consume	65 (34.0)	191
	1‒2×/week	58 (30.4)	
	3‒4×/week	31 (16.2)	
	5‒6×/week	3 (1.6)	
	Everyday	34 (17.8)	
**Seafood consumption (%)**	Do not consume	145 (75.9)	191
	1‒2×/week	41 (21.5)	
	3‒4×/week	5 (2.6)	
**Processed meat or sausage consumption (%)**	Do not consume	128 (67.0)	191
	1‒2×/week	33 (17.3)	
	3‒4×/week	12 (6.3)	
	5‒6×/week	2 (1.0)	
	Everyday	16 (8.4)	
**Smoking (%)**		19 (10.2)	187
**Metabolic Syndrome (%)**		122 (70.5)	173
**Cardiovascular Risk (%)**	Low	5 (2.7)	185
	Intermediate	17 (9.2)	
	High	126 (68.1)	
	Very high	37 (20.0)	
**IL-6, median (IQR)**		1.54 (1.2)	132
**IL-18, median (IQR)**		4.74 (4.8)	133
**MCP1, median (IQR)**		258.8 (195.8)	134
**Monocytes relative count, median (IQR)**		7.5 (3.2)	178

a Not informed or not available data were classified as missing data.

### Gout patients presented a higher intake of alcohol, uricogenic drinks, and had higher serum IL-6

Among variables potentially more prevalent in the gout group, male sex was more frequent compared to the non-gout group. Uricogenic foods were consumed more in the gout group, pointing to ethanol consumption (*p* = 0.006), sugary drinks intake (*p* = 0.001), as well as meat (*p* < 0.001), seafood (*p* < 0.001), and processed meat or sausage consumption (*p* = 0.034; Table 2). Uric acid was equally prevalent in the gout group compared to the non-gout group (*p* = 0.8), likely due to the use of urate-lowering therapy (Supplementary Table 2). In the inflammatory panel, IL-6 blood concentration was significantly higher in the gout group compared to the non-gout group (*p* = 0.019). Importantly, means are presented for descriptive purposes; however, non-parametric tests were applied for group comparisons when appropriate.

**Table 2:** Absolute and relative frequency and prevalence of Gout. Measurement units ‒ Uric acid: mg/dL, IL-18: pg/mL, and MCP1: pg/mL. Calculations were corrected by the Bonferroni method.

Characteristic	Non-gout (*n* = 91)	Gout (*n* = 102)^a^	p-value^b^
Sex			<0.001
Female	36 (82%)	8 (18%)	
Male	55 (37%)	94 (63%)	
Age, Mean (SD)	62.5 (12.8)	63.5 (12.5)	0.3
Uric acid, Mean (SD)	7.06 (1.81)	7.07 (2.13)	0.8
Body mass index, Mean (SD)	29.20 (4.97)	29.21 (5.16)	0.7
Physical Activity			0.013
Do not practice	62 (42%)	84 (58%)	
1‒2×/week	24 (59%)	17 (41%)	
>2×/week	4 (100%)	0 (0%)	
Alcoholic Beverages consumption			0.006
Do not consume	79 (53%)	71 (47%)	
1‒2×/week	11 (30%)	26 (70%)	
3‒4×/week	0 (0%)	1 (100%)	
Everyday	0 (0%)	3 (100%)	
Sugary Drinks consumption			0.001
Do not consume	44 (39%)	70 (61%)	
1‒2×/week	31 (74%)	11 (26%)	
3‒4×/week	6 (50%)	6 (50%)	
Everyday	9 (39%)	14 (61%)	
Red meat consumption			<0.001
Do not consume	11 (17%)	54 (83%)	
1‒2×/week	39 (67%)	19 (33%)	
3‒4×/week	20 (65%)	11 (35%)	
5‒6×/week	1 (33%)	2 (67%)	
Everyday	19 (56%)	15 (44%)	
Seafood consumption			<0.001
Do not consume	54 (37%)	91 (63%)	
1‒2×/week	32 (78%)	9 (22%)	
3‒4×/week	4 (80%)	1 (20%)	
Processed meat or sausage consumption			0.034
Do not consume	51 (40%)	77 (60%)	
1‒2×/week	21 (64%)	12 (36%)	
3‒4×/week	6 (50%)	6 (50%)	
5‒6×/week	1 (50%)	1 (50%)	
Everyday	11 (69%)	5 (31%)	
IL6	1.38 (0.90, 2.11)	1.85 (1.19, 3.08)	0.019
IL18	4.7 (2.2, 6.7)	5.0 (2.5, 7.7)	0.4
MCP1	256 (216, 405)	267 (223, 424)	0.4
Monocytes % (SD)	7.40 (6.20, 9.60)	7.55 (6.20, 9.20)	0.9

a Mean (SD); n (%).

b Wilcoxon rank sum test; Pearson’s Chi-Squared test; Fisher’s exact test.

### Sex, uricogenic food, and monocyte relative count are positively associated with gout

Several variables differed between gout and non-gout patients, while others expected to differ did not (Table 2). Importantly, to determine how independent variables were associated with gout, the adjustment for covariates was required. To address this issue, we compared three different models: 1) Univariate model: analyzing how each variable contributes to gout as an outcome, 2) Bivariate model: analyzing how each variable contributes to gout as an outcome, but this time, considering UA as a covariate, and 3) Multivariate model: analyzing how the combination of variables contribute to gout as an outcome (Table 3).

**Table 3:** Uni-, bi- and multivariate analysis from logistic regression of Gout with independent variables. Measurement units - Uric acid: mg/dL, IL-18: pg/mL, and MCP1: pg/mL. Univariate models represent crude associations. Bivariate models were adjusted for serum uric acid levels only. Multivariate models included all variables simultaneously, allowing the estimation of independent associations.

Characteristic	Univariate model			Bivariate model			Multivariate model		
	OR	95% CI	p-value	OR	95% CI	p-value	OR	95% CI	p-value
Uric acid	1.00	0.88, 1.15	>0.9	‒	‒	‒	1.07	0.83, 1.36	0.6
Sex	7.69	3.49, 18.9	<0.001	7.53	3.41, 18.5	<0.001	4.02	1.30, 14.5	0.022
Age	1.01	0.99, 1.04	0.3	1.02	0.99, 1.04	0.2	1.03	1.00, 1.07	0.086
Body mass index	1.00	0.93, 1.07	>0.9	1.00	0.93, 1.07	>0.9	1.03	0.94, 1.12	0.5
Physical activity	0.43	0.22, 0.80	0.009	0.39	0.19, 0.73	0.005	0.61	0.23, 1.50	0.3
Alcohol consumption	2.81	1.46, 6.12	0.005	2.85	1.48, 6.25	0.005	3.63	1.36, 11.8	0.021
Sugary drinks consumption	0.95	0.76, 1.18	0.6	0.95	0.76, 1.18	0.6	1.40	1.01, 1.96	0.046
Red meat consumption	0.70	0.56, 0.86	<0.001	0.70	0.56, 0.86	0.001	1.16	0.82, 1.63	0.4
Seafood consumption	0.20	0.09, 0.41	<0.001	0.21	0.10, 0.41	<0.001	0.45	0.17, 1.13	0.10
Processed meat or sausage consumption	0.73	0.56, 0.94	0.017	0.74	0.56, 0.94	0.020	0.78	0.52, 1.12	0.2
IL6	0.99	0.89, 1.06	0.8	0.99	0.88, 1.06	0.8	0.99	0.86, 1.06	0.8
IL18	1.04	0.97, 1.12	0.2	1.03	0.96, 1.11	0.4	1.01	0.92, 1.11	0.8
MCP1	1.00	1.00, 1.00	>0.9	1.00	1.00, 1.00	0.9	1.00	1.00, 1.00	0.9
Monocytes (%)	0.97	0.87, 1.09	0.6	0.97	0.87, 1.09	0.6	0.84	0.70, 0.98	0.029

CI, Confidence Interval; OR, Odds Ratio.

From univariate analysis (Table 3), we noticed that sex (OR = 7.69, *p* < 0.001), physical activity (OR = 0.43, *p* = 0.009), and alcohol consumption (OR = 2.81, *p* = 0.005) were significantly associated with gout, which corroborates previous.^16^^,^^17^^,^^18^^,^^19^ However, in the same model, some elements from the uricogenic diet appeared to be protective against gout - red meat (OR = 0.70, *p* < 0.001), seafood (OR = 0.2, *p* < 0.001) and processed meat and sausage consumption (OR = 0.73, *p* = 0.017) – which contradicts the actual literature.^20^^,^^21^ This paradoxical effect may be due to the small number of individuals in each category, which reduces the resolution of the data and obscures the true underlying pattern. A larger cohort with more observations per category, or still more categories, would likely provide clearer results and mitigate this issue.

In the second analysis, the bivariate model (Table 3), when uric acid was added as a covariate to the model, the same variables were identified as contributors to gout, indicating that uric acid did not modify the conclusions built in the first model. And, finally, in the third model, the multivariate model (Table 3), sex (OR = 4.28, *p* = 0.022), alcohol (OR = 3.63, *p* = 0.021), and sugary drinks consumption (OR = 1.40, *p* = 0.046) were found to contribute to gout development. However, these associations are modest and should be interpreted with caution. Additionally, in this same model, the monocyte relative count was significantly associated with gout as an outcome; in this case, having a higher number of circulating monocytes might be protective against gout (OR = 0.84, *p* = 0.029), which should be associated with the phagocytosis ability of these cells. Another explanation may lie in monocytes' ability to be primed in hyperuricaemia.^22^ However, here, none of the models could directly attribute the contribution of UA to gout, which may indicate the need to identify other factors that could help predict the prevalence and severity of the disease. Still, the use of urate-lowering therapies, especially by gout patients, might have masked UA contribution to this outcome (Supplementary Table 1).

### IL-6 is higher in gout and could predict gout flare frequency

**Fig. 1:**
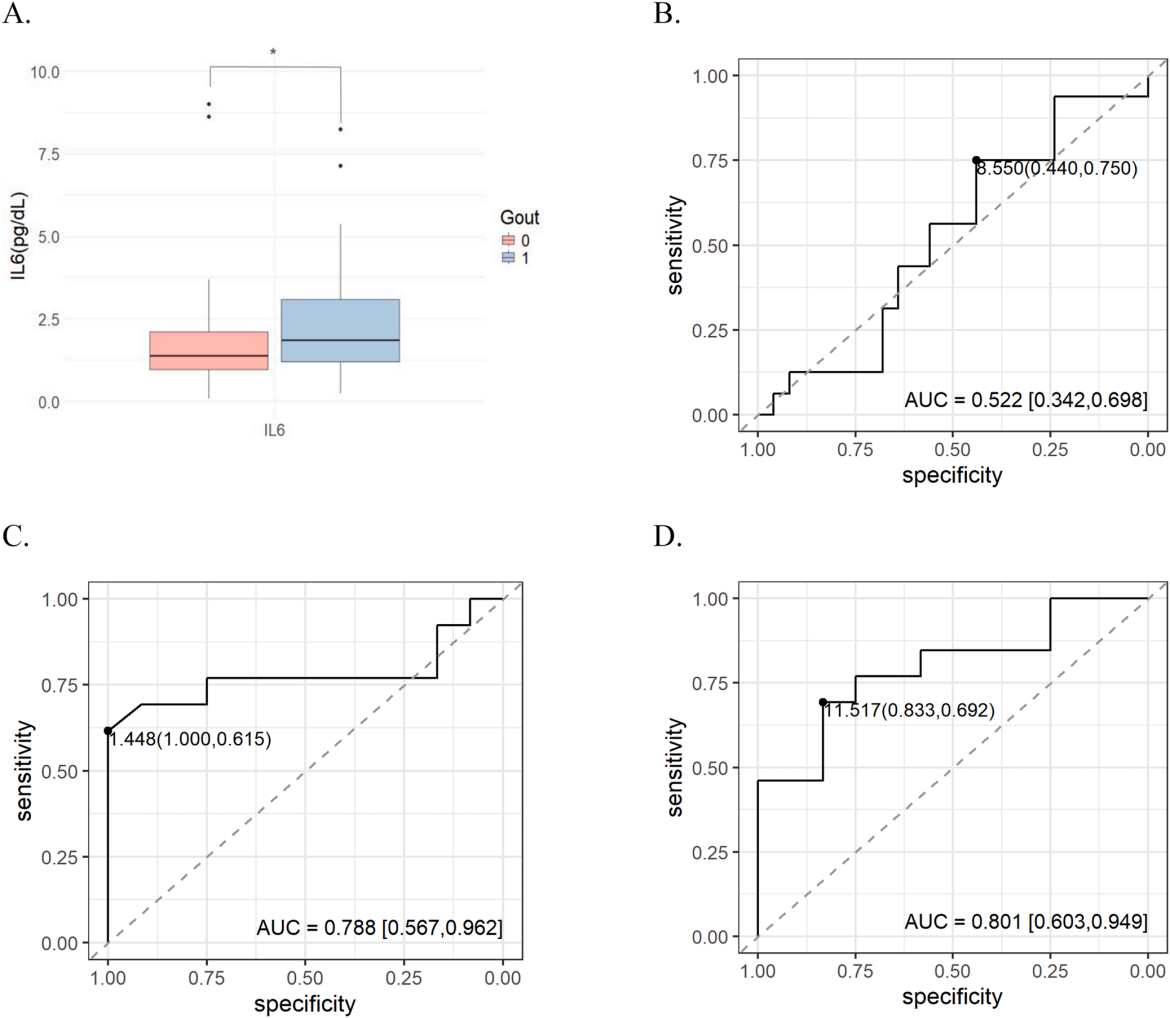
Cytokines between groups. (A) IL-6 levels in Gout vs. Non-Gout groups, being Gout = 1 and Non-Gout = 0; (B) Prediction of gout flare frequency based on UA concentration; (C) Prediction of gout flare frequency based on IL-6 concentration; (D) Prediction of gout flare frequency based on IL-6 concentration combined with monocyte count, the combination were obtained by multiplying the variable (*p < 0.05). In 1B the AUC=0.522 represents randomness. While 1C and 1D are considered not statistically different according to DeLong’s test (*p* = 0.8165).

Beyond the monocyte count, we investigated the inflammatory state of gout by assessing the serum concentration of cytokines and chemokines in each group (Supplementary Fig. 1). In this analysis, we observed that IL-6 levels were significantly higher in the gout group compared to the non-gout group (*p* = 0.018) (Fig. 1A), possibly indicating a chronic pro-inflammatory state in gout patients. Other cytokines were not different between gout and non-gout patients (Supplementary Fig. 1).

Building on the IL-6 association with gout, we further investigated whether IL-6 levels could serve as potential predictors of gout flare frequency. Patients experiencing high gout flare frequency (≥ 1 episode every 3-months) were compared to those with low frequency (< 1 episode every 3-months), and we found that IL-6 showed better discriminatory performance between the groups high and low frequency of gout flares (AUC = 0.78; Fig. 1C) than UA alone (AUC = 0.52; Fig. 1B). Predictive accuracy improved when combining IL-6 with relative monocyte count (AUC = 0.80; Fig. 1D), although the difference between the curves is not statistically significant according to DeLong’s test (*p* = 0.8165), both models retained good discriminative ability, and the addition of secondary variables ‒ especially those already commonly used in clinical practice ‒ could further improve the distinction between high and low flare frequency. Moreover, the superiority of IL-6 over uric acid in predicting flare frequency was further supported by the cross-validation model (Supplementary Fig. 2).

## Discussion

In this study, we conducted an observational analysis of the gout population in southern Brazil. We assessed data regarding biochemical and clinical parameters, lifestyle habits, gout flare frequency, and serum cytokine concentrations. These data were analyzed individually and in combination to infer risk factors associated with gout pathogenesis.

Patients with gout had a higher intake of uricogenic foods, including alcohol,^23^ fructose, particularly sugar-sweetened beverages,^24^ and meat,^20^ all of which are known to increase uric acid levels and gout risk.^25^ While meat consumption has been associated with a higher incidence of gout, vegetarian diets appear protective.^26^ Notably, as the study population was already receiving urate-lowering therapy, this may have attenuated the observed associations between gout and hyperuricemia.

Deepening the analysis towards the inflammatory cues that might be associated with gout, we found that the monocyte relative count is associated with gout. In this regard, urate has been reported to enhance the pro-inflammatory response and alter circulating monocyte counts,^27^ although urate was stated as inducing pyroptosis in macrophages.^28^ In this sense, UA has been described as a disruptive factor of immune cell function,^15^ and the cell ratio was able to distinguish ill from healthy patients regarding the inflammatory context.^29^ Macrophage hypophagia has been proposed as a mechanism of immune exhaustion in antibody-dependent cellular phagocytosis^30^ and could explain the resistance of tophi to dissolving found in chronic gout. In this context, a higher proportion of monocytes may also reflect a potential protective role of the measured parameter in the non-gout group (OR< 1); however, further studies are needed to investigate possible confounding factors, such as medication use. Also, the role of monocyte count in predicting an increased risk of gout flares should be interpreted with caution, as although the AUC increased from 0.788 (IL-6 alone) to 0.801 (IL-6 × monocytes), more robust analyses showed no significant difference between the models (DeLong *p* = 0.8165).

In addition to macrophages' involvement, cytokines have been proposed as biomarkers of gout. Prior studies in the Chinese population easily differentiated gout from healthy controls based on serum cytokines,^27^ in which elevated levels of cytokines ‒ particularly IL-1β, IL-6, IL-8, and TNF-α ‒ were observed during flares. In the present study, only IL-6 was associated with a higher likelihood of gout flares in the intercritical phase. It is important to notice that IL-6 did not retain an independent association with gout diagnosis in the multivariate model, despite the initial bivariate finding. Other cytokines, such as IL-1β, IL-18, and TNF-α, were also reported in the gout flare episodes.^31^^,^^32^ This explains the low or undetectable levels of some cytokines in this study, IL-1β or TNF-α, possibly due to measurement during the intercritical period or assay limitations. However, TNF-α has been detected in intercritical gout patients in recent studies,^12^ as well as IL-10, which was higher in gout patients than in healthy controls,^27^ contrasting the present results with some previous reports and highlighting the need for further investigation, particularly considering sample size, biological variability, and timing of collection.

The main limitations of the present study include the modest sample size. Although all eligible patients from the outpatient clinic who provided informed consent and did not meet any exclusion criteria were included, the overall sample size remained below the ideal threshold. Additionally, the study was a cross-sectional and single-center design, which limits causal inference and generalizability. Also, dietary data were self-reported, making them prone to recall and social desirability bias. Urate-lowering therapy was not adjusted in all models and may have confounded uric acid-related findings. And, finally, cytokines were measured at a single time point outside flare episodes, which may explain the low detection of IL-1β and TNF-α. Overall, the analyses would benefit from larger cohorts to strengthen conclusions regarding the inflammatory profile of gout and potential predictors of flares. Finally, the present analysis of a Brazilian gout population contributes to the limited epidemiological data on gout in South America, as well as suggests that serum IL-6 and monocyte relative may serve as possible predictors of gout and gout flare frequency.

## Conclusion

This observational study analyzed a Brazilian population of gout patients and compared them with non-gout patients. Although UA is currently highly used in the clinical approach to gout, we suggest that other inflammatory components should be considered to better understand gout pathogenesis, such as relative monocyte count and IL-6. Importantly, the findings presented here are based on an observational study and will require validation in independent cohorts, longitudinal follow-ups, or experimental studies to confirm their clinical relevance. Last, these findings could contribute to a better understanding of disease heterogeneity in patients with gout, support early diagnosis, and improve the quality of life of patients undergoing or potentially susceptible to gout.

## Declaration of generative AI and AI-assisted technologies in the writing process

Generative-AI was used in this manuscript to improve language and readability. The authors reviewed all revisions and take full responsibility for the final manuscript.

## CRediT authorship contribution statement

**Jordana Dinorá de Lima:** Conceptualization, Investigation, Methodology, Formal analysis, Data curation. **Leonel Witcoski Junior:** Writing – original draft, Writing – review & editing. **André Guilherme Portela de Paula:** Investigation, Methodology, Data curation, Writing – original draft. **Andressa Pacheco Czaikovski:** Investigation, Data curation. **Bruna Sadae Yuasa:** Investigation, Data curation. **Gabriela Yanaze Takamatsu:** Investigation, Data curation. **Caio Cesar Souza Smanioto:** Investigation, Data curation. **Maria Clara da Cruz Silva:** Investigation, Data curation. **Ederson Borges Francisco:** Investigation, Data curation. **Juliana Navarro Ueda Yaochite:** Supervision, Methodology, Formal analysis, Resources. **Marco Antonio de Freitas Clementino:** Methodology, Formal analysis. **Felipe Fortino Verdan:** Data curation. **Valéria Vidal:** Data curation. **Cyntia Nancy Weçoski:** Data curation. **Niels Olsen Saraiva Câmara:** Supervision, Resources. **Fellype Barreto:** Methodology, Conceptualization, Supervision, Resources. **Eduardo S. Paiva:** Conceptualization, Supervision, Resources. **Valderílio Feijó Azevedo:** Conceptualization, Supervision, Resources. **Bianca de Oliveira Cata-Preta:** Methodology, Supervision, Formal analysis, Resources. **Tárcio Teodoro Braga:** Conceptualization, Supervision, Project administration, Resources, Writing – review & editing.

## Declaration of competing interest

The authors declare no conflicts of interest.

## Data Availability

The datasets generated and/or analyzed during the current study are available from the corresponding author upon reasonable request.

## Appendix Supplementary materials

Image, application 1
