# Supplementary material for: Clinical and inflammatory factors associated with prevalent gout in a Brazilian hospital population
Source: Clinics (Sao Paulo). 2026 Aug 11;81:101083. doi: 10.1016/j.clinsp.2026.101083 (PMC13487591; doi:10.1016/j.clinsp.2026.101083)
Supplement: Supplementary file 1 [file mmc1.docx]

**CLINICS-D-25-01181**

**Supplementary Material**

**Possible confounding factors**

In addition to the analyzing exploring the association between serum uric acid and the presence of gout, we included covariates that could potentially confound or mask this relationship. These variables were selected based on prior literature linking them to gout risk: sex and age (Harrold et al., 2006; Wallace et al., 2004), body mass index (Karlsson et al., 2023), physical activity frequency (Gwinnutt et al., 2022), alcohol consumption (Lyu et al., 2024), sugary drinks consumption (Choi & Curhan, 2008), red meat and seafood consumption (Choi et al., 2005), processed meat or sausage consumption (Xia et al., 2018), IL-6 (Zhang et al., 2025), IL-18 (Martinon et al., 2006), MCP1 serum measurements (Chang et al., 2025), as well as number of monocytes (Grainger et al., 2013). Unfortunately, despite the association between gout and IL1β (Martinon et al., 2006; Terkeltaub et al., 2009), we did not have enough detectable serum IL1β measurements to explore this association or use this variable as a covariate. These covariates were selected based on their previously reported associations with gout. Notably, except for physical activity, all variables have been positively associated with gout in earlier studies. Alcohol, sugar-sweetened beverages, red meat, seafood, and processed meats were considered uricogenic foods, as they provide substrates such as fructose and purines that contribute to uric acid synthesis. Moreover, a limitation of this study is that urate-lowering therapy was not accounted for in the analyses. While such treatments may affect serum uric acid concentrations and partially explain the absence of differences between inflammatory profiles, comprehensive data on treatment exposure (including dosage, duration, adherence, and treatment effectiveness) were not available for all participants. Consequently, adjustment for medication use was not performed, and residual confounding related to urate-lowering therapy cannot be excluded.

**Supplementary Table 1** Comorbidities along with the sampled groups.

| **Characteristic** | **Non-gout (n=91)^a^** | **Gout (n=102)^a^** | **p-value^b^** |
| --- | --- | --- | --- |
| Dyslipdemia | 57 (45%) | 70 (55%) | 0.4 |
| High Blood Pressure | 76 (46%) | 91 (54%) | 0.2 |
| Type 2 Diabetes | 40 (56%) | 32 (44%) | 0.071 |
| Heart Failure | 9 (41%) | 13 (59%) | 0.5 |
| Coronary Artery Disease | 14 (35%) | 26 (65%) | 0.084 |
| Stroke | 9 (41%) | 13 (59%) | 0.5 |
| Thrombosis | 11 (50%) | 11 (50%) | 0.8 |
| Arrythmia | 4 (24%) | 13 (73%) | 0.041 |
| Nephrolithiaiss | 24 (60%) | 16 (40%) | 0.016 |
| Chronic Kidney Disease | 60 (55%) | 49 (45%) | 0.009 |
| Hypothyroidism | 14 (52%) | 13 (48%) | 0.6 |

^a^ n (%).

^b^ Pearson’s Chi-Squared test; Fisher’s exact test.

**Supplementary Table 2** Medication use in the sampled population.

| **Characteristic** | **Non-gout (n=94)^a^** | **Gout (n=102)^a^** | **p-value^b^** |
| --- | --- | --- | --- |
| Colchicine | 0 (0%) | 39 (100%) | <0.001 |
| Diuretic | 57 (66%) | 30 (34%) | <0.001 |
| Allopurinol | 6 (6.8%) | 82 (93%) | <0.001 |
| Statin | 55 (51%) | 52 (49%) | 0.2 |
| Antihypertensives | 79 (47%) | 90 (53%) | 0.6 |
| Antiagregant | 26 (47%) | 29 (53%) | >0.9 |
| Fibrate | 7 (54%) | 6 (46%) | 0.6 |
| Beta-blocker | 33 (50%) | 33 (50%) | 0.6 |
| Vasodilator | 14 (58%) | 10 (42%) | 0.2 |
| Anticoagulant | 3 (21%) | 11 (79%) | 0.045 |
| Hypoglycemic | 32 (52%) | 30 (48%) | 0.4 |

^a^ n (%).

^b^ Pearson’s Chi-squared test; Fisher’s exact test.

**Supplementary Figure 1 Serum cytokines between gout and non-gout.** Aiming to represent all cytokines in the same scale, all values were log10-transformed ‒ Gout: 1 vs. Non-Gout: 0 analysis.

**Supplementary Figure 2 Cross-validation of predictive models.** (A) AUC for gout flare frequency based on UA (kappa_best_accuracy_ = 0.00); (B) AUC for gout flare frequency based on IL-6 (kappa_best_accuracy_ = 0.7). (C) Multiple regression model predicting gout presence (kappa_best_accuracy_ = 0.2).

**Appendix**

**Supplemental Material Appendix 1** Free and informed Consent Form

**TERMO DE CONSENTIMENTO LIVRE E ESCLARECIDO**

Nós, Prof. Tárcio Teodoro Braga, Caio Cesar Souza Smanioto e Maria Clara da Cruz Silva (alunos do curso de Medicina ‒ UFPR), Profa. Angélica Beate Winer Boldt, Profa. Karin Braun Prado e o Doutor Valderílio Feijó Azevedo, pesquisadores da Universidade Federal do Paraná, estamos convidando o senhor(a), paciente frequentador(a) do Serviço de Reumatologia do Complexo Hospital de Clínicas da UFPR, a participar de um estudo intitulado “VARIAÇÕES GENÉTICAS EM NAIP E SUAS CORRELAÇÕES COM A HIPERURICEMIA E A GOTA”. Vamos estudar o papel de um gene (NAIP) na gota. Esse estudo será importante para entender melhor como o corpo humano reage ao ácido úrico alto e porque algumas pessoas desenvolvem a gota e outras não. O conhecimento adquirido com esse estudo poderá ajudar a desenvolver, por exemplo, novos tipos de tratamento para a doença.

O objetivo desta pesquisa é coletar alguns dados como idade, peso, altura, medida da cintura, pressão arterial, histórico de doenças pessoal e familiar, laboratoriais (ácido úrico, ureia, creatinina e perfil lipídico) e de análises do gene NAIP (gene alvo da pesquisa), em pacientes com gota, com ácido úrico alto, mas sem gota ou com ácido úrico normal. A análise dos dados coletados poderá ajudar a responder algumas perguntas importantes a respeito da gota e permitirá verificar se o gene NAIP pode estar envolvido com o desenvolvimento da doença. Além disso, a pesquisa pretende identificar problemas relacionados com a gota (como pressão alta, colesterol alto e diabetes) nos pacientes participantes e, isso poderá ajudar a pensar em maneiras de prevenção e tratamento dessas condições.

Caso o senhor(a) participe da pesquisa, será necessário realizar uma entrevista em que algum dos pesquisadores irá aplicar um questionário contendo perguntas sobre seu histórico de doenças e de sua família, medicamentos utilizados, sobre alguns hábitos de vida (alimentação, exercícios físicos) e realizará algumas medidas (altura, peso, medida da cintura e pressão). Além disso, será realizada a coleta de uma amostra de 25 mL (cerca de duas colheres de sopa) de sangue, em uma veia do braço, como as realizadas para exames de sangue de rotina. A coleta será realizada por um profissional capacitado e experiente. Esse sangue será utilizado para exames de laboratório citados anteriormente e também para extração de material genético para as análises do gene NAIP, como descrito no parágrafo anterior.

A realização da entrevista pelo pesquisador e coleta da amostra sanguínea levará aproximadamente trinta (30) minutos (meia hora) e ocorrerá no Complexo Hospital de Clínicas da Universidade Federal do Paraná (CHC-UFPR. R. Gen. Carneiro, 181 ‒ Alto da Glória, Curitiba ‒ PR, 80060-900). Eventualmente, poderá ser realizada no Centro de Pesquisas Edumed (R. Bpo. Dom José, 2495 ‒ Seminário, Curitiba ‒ PR, 80440-080).

É possível que senhor(a) experimente algum desconforto, principalmente relacionado à coleta de sangue. Pode haver constrangimento ou desconforto nas respostas às questões feitas pelos pesquisadores. Caso ocorra qualquer problema após a entrevista e a coleta, o participante poderá entrar em contato com os pesquisadores.

Os benefícios esperados com essa pesquisa são: maior entendimento sobre a gota, o motivo de algumas pessoas desenvolverem a gota e outras não, e qual o papel do gene NAIP na doença. Além de melhor ideia a respeito de problemas cardiovasculares relacionadas com o ácido úrico alto. Embora nem sempre (o Senhor, a Senhora) seja diretamente beneficiado(a) por sua participação neste estudo, os resultados podem contribuir para todos os pacientes com gota e a sociedade se forma geral.

Os pesquisadores Tárcio Teodoro Braga (professor), Caio Cesar Souza Smanioto e Maria Clara da Cruz Silva (alunos de Medicina-UFPR), responsáveis por este estudo, poderão ser localizados para esclarecer eventuais dúvidas que o senhor(a) possa ter e fornecer-lhe as informações que queira, antes, durante ou depois de encerrado o estudo. O contato poderá ser feito por e-mail (tarcio.braga@ufpr.br, caiosmanioto@ufpr.br ou mariaclaracruz@ufpr.br) telefone em horário comercial (telefone 041 33611691 - das 8:00 às 18:00). Em situações de emergência ou urgência, relacionadas à pesquisa, os mesmos poderão ser contatados pelo telefone (número de telefone disponível nas 24 horas, com acesso direto com o pesquisador envolvido – 041 99767-4663).

A sua participação neste estudo é voluntária e se senhor(a) não quiser mais fazer parte da pesquisa, poderá desistir a qualquer momento e solicitar que lhe devolvam este Termo de Consentimento Livre e Esclarecido assinado. A desistência de participação desta pesquisa não irá interferir em seu direito em continuar atendimento/tratamento habituais no Complexo Hospital de Clínicas da UFPR.

As informações relacionadas ao estudo poderão ser conhecidas por pessoas autorizadas como os pesquisadores envolvidos. No entanto, se qualquer informação for divulgada em relatório ou publicação, será feito sob forma codificada, para que a sua identidade seja preservada e seja mantida a confidencialidade.

O material obtido (sangue coletado e dados recolhidos com a entrevista) será utilizado unicamente para esta pesquisa e será destruído ou descartado ao término do estudo, dentro de dois anos.

As despesas necessárias para a realização da pesquisa (coleta das amostras, análises laboratoriais e genéticas) não são de sua responsabilidade, então o senhor(a) não terá nenhum custo e não deverá pagar nada para participar da pesquisa.

O senhor(a) terá a garantia de que problemas como: tonturas ou desmaios relacionados à coleta de sangue para o estudo, serão tratados no local da coleta pelos responsáveis envolvidos no procedimento. Caso o senhor(a) necessite se deslocar até o Centro de Pesquisas Edumed unicamente para a realização da entrevista e coleta de sangue para a pesquisa, os pesquisadores se comprometem com o ressarcimento dos gastos com o deslocamento até o local.

Quando os resultados forem publicados, não aparecerá seu nome, e sim um código.

Se você tiver dúvidas sobre seus direitos como participante de pesquisa, você pode contatar também o Comitê de Ética em Pesquisa em Seres Humanos (CEP/SD) do Setor de Ciências da Saúde da Universidade Federal do Paraná, pelo telefone 3360-7259. O Comitê de Ética em Pesquisa é um órgão, que existe nas instituições que realizam pesquisa envolvendo seres humanos no Brasil e foi criado com o objetivo de proteger os participantes de pesquisa, em sua integridade e dignidade, e assegurar que as pesquisas sejam desenvolvidas dentro de padrões éticos (Resolução nº 466/12 Conselho Nacional de Saúde).

Eu, ______________________________________________________________ li esse Termo de Consentimento e entendi sobre o que se trata esse estudo que concordei em participar. A explicação que recebi menciona os riscos e benefícios. Eu entendi que sou livre para interromper minha participação a qualquer momento sem justificar minha decisão e sem qualquer prejuízo para mim nem para meu tratamento no Complexo Hospital de Clínicas. Eu entendi o que não posso fazer durante a pesquisa e fui informado que serei atendido sem custos para mim se eu apresentar algum problema diretamente relacionado ao desenvolvimento da pesquisa (como disposto no item l).

Eu concordo voluntariamente em participar deste estudo.

______________________________________________

Assinatura do Participante e/ou Responsável Legal

______________________________________________

Assinatura do Pesquisador e/ou quem aplicou o TCLE

Curitiba,

**Supplemental Appendix 2** In-person survey.

**Questionário sobre história clínica e hábitos de vida – Projeto Naip e gota**

Grupo: ( ) 1 - Paciente com gota ( ) 2 - Hiperuricêmico sem gota ( ) 3 - Normouricêmico

Código do paciente (nº):

Nome:

Endereço:

Telefone:

E-mail:

Data de aplicação do questionário: ___ /___ /___

Aplicador:

Informações gerais

Data de nascimento (idade): ___ /___ / ___ (___ anos)

Sexo: ( ) M ( ) F

Local de nascimento:

Idade da menopausa: ( ) Não se aplica ( ) Se aplica:

Cor ou raça (autodeclarada): ( ) Branca ( ) Preta	( ) Parda	( ) Amarela	( ) Indígena

Ancestralidade (ascendência específica): ( ) Europeus ( ) Asiáticos ( ) Africanos ( ) Ameríndios ( ) Árabes ( ) Judeus ( ) Outros ______________________________

Histórico de doenças (perguntar se paciente tem ou já teve alguma das condições)

Doenças cardiovasculares e associadas: ( ) Sim ( ) Não

De qualquer forma, confirmar perguntando sobre algumas condições específicas:

( ) HAS - “pressão alta” ( ) coronariopatias - infarto, angina (“dor no peito”) ( ) insuficiência cardíaca – “coração fraco” ( ) AVE – “derrame” ( ) trombose ( ) DAOP - claudicação intermitente (dor nas pernas quando começa a caminhar) ( ) Diabetes mellitus - “glicose alta no sangue” ( ) Dislipidemia – “colesterol alto”

Em caso afirmativo, especificar:

Qual(is) a(s) doença(s)? _______________________________________________

Qual idade de diagnóstico? ________________________________________

Realizou procedimento cirúrgico relacionado? Quando? _________________

Realiza acompanhamento médico? __________________________________

Realiza algum tipo de tratamento? __________________________________

Doenças reumatológicas e autoimunes: ( ) Sim ( ) Não

De qualquer forma, confirmar perguntando sobre algumas condições específicas:

( ) GOTA ( ) Artrite reumatoide ( ) Artrose ( ) Lúpus eritematoso sistêmico ( ) Psoríase ( ) Artrite psoriática ( ) Espondilite anquilosante ( ) Esclerodermia ( ) Febre reumática ( ) Osteoporose ( ) Bursites ( ) Tendinites ( ) Polimialgia reumática ( ) Outra: _________

Em caso afirmativo, especificar:

Qual(is) a(s) doença(s)? ________________________________________________

Qual idade de diagnóstico? ________________________________________

Realizou procedimento cirúrgico relacionado? Quando? _________________

Realiza acompanhamento médico? __________________________________

Realiza algum tipo de tratamento? __________________________________

Em caso específico de GOTA, detalhar:

Como descobriu? ________________________________________________

Possui forma crônica (tofos)? Há quanto tempo? _______________________

Possui nefrolitíase associada? Há quanto tempo? _______________________

Nota algum fator associado (desencadeante) às crises? __________________

Algum fator melhora as crises? Quanto tempo elas duram? ______________

Gostaria de acrescentar mais alguma informação? ______________________

Outras doenças

Doenças renais: ( ) Sim ( ) Não

Em caso negativo, perguntar especificamente sobre nefrolitíase (pedra no rim) e insuficiência renal;

Em caso afirmativo, especificar:

Qual(is) a(s) doença(s)? ___________________________________________

Qual idade de diagnóstico? ________________________________________

Realizou procedimento cirúrgico relacionado? Quando? _________________

Realiza acompanhamento médico? __________________________________

Realiza algum tipo de tratamento? __________________________________

Doenças de outros órgãos / sistemas: ( ) Sim ( ) Não

Em caso afirmativo, especificar:

Qual(is) a(s) doença(s)? ___________________________________________

Qual idade de diagnóstico? ________________________________________

Realizou procedimento cirúrgico relacionado? Quando? _________________

Realiza acompanhamento médico? __________________________________

Realiza algum tipo de tratamento? __________________________________

Histórico familiar de doenças (perguntar sobre pais, avós, irmãos, tios, filhos e netos)

Doenças cardiovasculares (considerar as mesmas perguntadas anteriormente): ( ) Sim ( ) Não

Em caso afirmativo, especificar:

Quais os familiares? ______________________________________________

Qual(is) a(s) doença(s)? ___________________________________________

Doenças reumatológicas e autoimunes (considerar as mesmas perguntadas anteriormente): ( ) Sim ( ) Não

Em caso afirmativo, especificar:

Qual(is) os familiares? _____________________________________________

Qual(is) a(s) doença(s)? ___________________________________________

Em caso específico de histórico familiar positivo para GOTA, especificar (se souber):

Como descobriu? Qual a idade do diagnóstico? ________________________

Possui forma crônica (tofos)? Há quanto tempo? _______________________

Possui nefrolitíase associada? Há quanto tempo? _______________________

Nota algum fator associado (desencadeante) às crises? __________________

Algum fator melhora as crises? Quanto tempo elas duram? ______________

Gostaria de acrescentar mais alguma informação? ______________________

Medicamentos em uso

Perguntar especificamente por: ( ) Diuréticos (furosemida, bumetanida, hidroclorotiazida, clortalidona) ( ) Ácido acetilsalicílico (AAS, aspirina) ( ) Complexo vitamínico contendo niacina (vitamina B3) ( ) Imunossupressores (ciclosporina) ( ) Antibióticos (etambutol, pirazinamida)

Listar medicamentos em uso (se possível, incluir posologia e tempo de uso):

Hábitos de vida

Pratica atividades físicas? ( ) Sim ( ) Não Com que frequência? ________________

Ingere bebidas alcoólicas? ( ) Sim ( ) Não Com que frequência? ________________

Em caso afirmativo, listar as bebidas consumidas: ______________________

Ingere bebidas açucaradas (refrigerantes, achocolatados, sucos etc)? ( ) Sim ( ) Não

Com que frequência? ___________________________________________________

Consome carnes vermelhas? ( ) Sim ( ) Não Com que frequência? _______________

Consome frutos do mar? ( ) Sim ( ) Não Com que frequência? _______________

Consome alimentos com alto teor de açúcares (bolos, pudins, doces em geral)?

( ) Sim ( ) Não Com que frequência? ____________________________________

Consome alimentos embutidos (presunto, salsichas, salames etc) ? ( ) Sim ( ) Não

Com que frequência? ___________________________________________________

Como costuma ser sua alimentação no dia a dia? _____________________________

Nota alguma associação entre hábito alimentar ou outros hábitos de vida e crises de gota? ________________________________________________________________

Dados antropométricos e PA (pedir permissão do paciente para algumas aferições)

Altura: _________ metros

Peso: __________ quilogramas

Calcular IMC: _________ kg/m²

( ) Menor que 18,5 – baixo peso

( ) Entre 18,5 e 24,9 – peso normal

( ) Entre 25 e 29,9 – sobrepeso

( ) Entre 30 e 34,9 – obesidade classe I

( ) Entre 35 e 39,9 – obesidade classe II

( ) Acima de 40 – obesidade classe III

Circunferência abdominal: ________ cm

Pressão arterial (aferida em braço direito, com paciente sentado): ________ mmHg;
